# Growth and Development Responses of the Rhizome-Root System in *Pleioblastus pygmaeus* to Light Intensity

**DOI:** 10.3390/plants11172204

**Published:** 2022-08-25

**Authors:** Weiwei Huang, Yongyan Ding, Shucong Wang, Chao Song, Fusheng Wang

**Affiliations:** 1 Co-Innovation Center for Sustainable Forestry in Southern China, Nanjing Forestry University, 159 Longpan Road, Nanjing 210037, China; 2 Bamboo Research Institute, Nanjing Forestry University, 159 Longpan Road, Nanjing 210037, China; 3 Department of Geosciences and Natural Resource Management, The University of Copenhagen, Rolighedsvej 23, DK-1958 Frederiksberg, Denmark; 4 College of Biology and the Environment, Nanjing Forestry University, 159 Longpan Road, Nanjing 210037, China; 5 College of Field Engineering, Army Engineering University of PLA, 88 Houbiaoying Road, Nanjing 210001, China

**Keywords:** dwarf bamboo, light conditions, morphological features, growth pattern

## Abstract

Light, as a primary source of energy, directly or indirectly influences virtually all morphological modifications occurring in both shoots and roots. A pot experiment was conducted to assess the growth patterns of one-year-old *Pleioblastus pygmaeus* plants’ rhizome-root systems and their responses to different light intensities from 11 March to 26 December 2016. The experiment design scheme was 3.87% (L1), 11.25% (L2), 20.25% (L3), 38.76% (L4), 60.70% (L5), and 100% full sunlight (control CK). The results indicated that along the growing period from March to December, eight of the eleven studied parameters of the rhizome-root system showed significant variability and diverse growth patterns. In addition, light intensity is a key factor for determining *P. pygmaeus* plants’ rhizome and root growth. Specifically, the light intensity had a significant, positive, and linear/or almost linear impact on the number of old and new rhizomes, old rhizome length, new rhizome diameter, as well as the culm root diameter. A nonlinear and positive relationship was found between light intensity and the listed three parameters, i.e., new rhizome length, new rhizome internode length, and rhizome root length. The value of the above-mentioned three parameters significantly increased when affected from 0% to 40–60% of full sunlight and then gradually increased until 100% of full sunlight. The ratio of aboveground dry weight to underground dry weight (A/U ratio) showed a single peak curve with increasing light intensity and presented the highest value under ca. 55% full sunlight. Furthermore, 40% full sunlight (equal to an average light of 2232 lux) might be the threshold for *P. pygmaeus* rhizome-root system growth. When the light intensity was below 40%, the generalized additive models (GAMs) predicted value of most studied parameters decreased to lower than zero. In conclusion, current study provides a solid basis for understanding the dynamic growth and development of *P. pygmaeus* rhizome-root system, and its responses to different light conditions, which could be used as inputs to *P. pygmaeus* plant cultivation.

## 1. Introduction

Bamboo has approximately 1300 species under 150 genera worldwide and is distributed across a wide range of tropical and subtropical areas, from alluvial plains to high mountains [1,2]. China has more than 500 bamboo species in 35 genera, accounting for 46% of the world’s bamboo species [3]. *Pleioblastus pygmaeus* (Miq.) Nakai is a dwarf bamboo which prefers to grow in moderate moisture and well-drained soil, and was introduced from Japan to China in the early 20th century. *P. pygmaeus* is an excellent ground cover ornamental plant, which has slender and erect green stalks, purplish nodes, and palm-shaped and emerald green leaves. In addition, for its well-developed and intricate rhizome-root system, it shows a strong water and fertilizer retention. Nowadays, the application demand for *P. pygmaeus* in landscaping is continually increasing in China, so its economic value is also getting an increase.

Light, as a primary source of energy, is one of the most important environmental factors for plant growth and survival. In the presence of light, the photosynthesis of green plants combines carbon dioxide and water to form carbohydrates and oxygen [4,5]. In order to survive and grow in a range of light conditions, plants dynamically adjust their architecture to optimize growth and performance in response to different light intensity [6]. A variety of photomorphogenic responses have been investigated extensively in the aboveground parts of plants [7,8,9,10,11,12]. Under deep shade, plants reduce their leaf dry matters and stem diameters as well as photosynthetic rate [7,8,9,13,14]. However, under low light intensity, tolerant species try to increase stem height and leaf area in order to increase the ability of capturing light and the net assimilation rate [15,16,17,18]. Under natural growth conditions, aboveground parts are directly exposed to light whereas root systems develop underground, shielded from direct illumination [19]. Notably, accumulating evidence demonstrates that underground roots are able to directly or indirectly perceive light signals to trigger photomorphogenic responses and experience dramatic changes in morphology and development under changing light conditions [6,9,14,19,20,21]. Concerning *P. pygmaeus*, as an excellent ground cover ornamental plant for soil consolidation and slope protection, it is very important to understand the responses of its rhizome-root morphological and developmental characteristics to changing light conditions [22].

Based on the rhizome growth patterns, bamboos were divided into three main types: the amphipodial, monopodial, and sympodial [23,24]. *P. pygmaeus* has an amphipodial rhizome, which processes a mixed culm morphology both aggregated and scattered. The detailed features are: (i) some of the underground rhizomes are sympodial with short rhizomes and internodes, and cannot spread in soil for a long distance; top buds unearth to generate new culms and stalks grow in dense clusters. (ii) The other part of the underground system is monopodial with stems spreading underground called “whipper root”; lateral buds unearth to form new culms and the stalks are scattered. Rhizomes together with roots generated from rhizome nodes form a rhizome-root system which have a decisive role in bamboo growth [25,26]. The rhizome-root system functions in food storage, fluid transport, and vegetative reproduction [27]. Young bamboos start to grow the new culms in height first, and then expand branches and leaves. Therefore, there is little leaf photosynthesis by new bamboo shoot systems during its culm height growth. Rhizome-root systems widely spread and connect the young culms with other mature bamboo culms to transport carbohydrates and nutrients for new culm height growth [27,28,29,30]. A strong translocation function of the *Phyllostachys pubescens* Mazel ex H. de Lehaie rhizome was that more than 20% of the compensative water used during summer was transferred from older culms through connected rhizomes [31]. As well as the carbohydrate storage and fluid transport function, a reticular and extensive underground rhizome-root system of bamboo can effectively bind and hold topsoil against soil erosion [2,32,33,34].

Very limited published evidence exists in the area of bamboo rhizome-root system responses to different light intensities. Our previous study found that low light intensity decreased underground dry weight, number of all rhizomes, mean length, and diameter of rhizomes of *P. pygmaeus* [22]. However, limited attention has been given to fully understanding the growth status of bamboo rhizomes and roots under changing light conditions. Thus, a pot experiment was conducted to assess the temporal dynamic growth of *P. pygmaeus* old and new rhizomes, culm roots, and rhizome roots during growing season from April to December, and to examine how the growth of its rhizome-root system is affected by different light intensity.

## 2. Results

### 2.1. Light Intensity Variation

**Figure 1:**
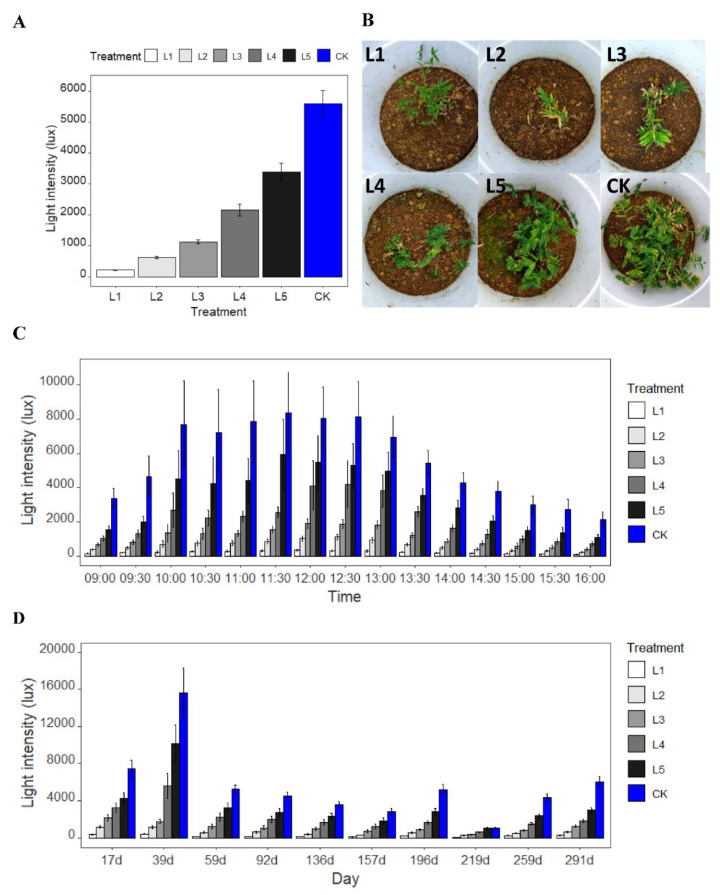
The plots of (**A**) mean light intensity under six light levels; (**B**) typical phenotype of *P. pygmaeus* on November 30, 2016; (**C**) diurnal variation of light intensity under six light levels; (**D**) light intensity along the investigation time under six light levels. The average light intensity of different treatments is L1 = 3.87%, L2 = 11.25%, L3 = 20.25%, L4 = 38.76%, L5 = 60.70%, and CK = 100% of full sunlight.

Across all the survey times, the average light intensity was decreased corresponding to the decreasing aperture of the cover plates, showing CK > L5 > L4 > L3 > L2 > L1 (Figure 1A). For the diurnal variation, the light intensity increased from 9:00 o’clock, then reached the maximum value between 11:30 and 12:30 o’clock, and thereafter decreased until the last records at 16:00 o’clock (Figure 1C). The maximum average light illumination of control (CK) during a day was 8372.73 lux, which was 25.10, 7.56, 4.41, 1.99, and 1.41 times higher than that of treatment L1, L2, L3, L4, and L5, respectively. In addition, the minimum mean light intensity of the control (CK) was 2135.87 lux, which was 21.97, 9.46, 5.28, 2.96, and 1.97 times higher than treatment L1, L2, L3, L4, and L5, respectively.

During the treatment from April to December in 2016, the light intensity of treatment L1, L4, L5, and CK firstly increased from 17 d to 39 d, reaching the maximum value, and then decreased and reached the minimum value at 219 d, and thereafter increased again (Figure 1D). As the investigation time continued, the light intensity of treatment L2 and L3 decreased from 17 d, reaching the minimum value at 219 d, and thereafter increased until the last records at 291 d. At 39 d, the light illumination of control (CK) was 15,619.33 lux, which was 39.03, 13.50, 8.84, 2.79, and 1.54 times higher than that of treatment L1, L2, L3, L4, and L5, respectively. At 219 d, the light illumination of the control (CK) was 1046.73 lux, which was 11.80, 3.51, 2.72, 1.71, and 0.99 times larger than that of treatment L1, L2, L3, L4, and L5, respectively.

Overall, the average illumination intensity under treatment L1, L2, L3, L4, and L5 was 3.87%, 11.25%, 20.25%, 38.76%, and 60.70% of the control (CK), respectively.

### 2.2. Rhizome and Root Growth Variation during Growing Period

**Figure 2:**
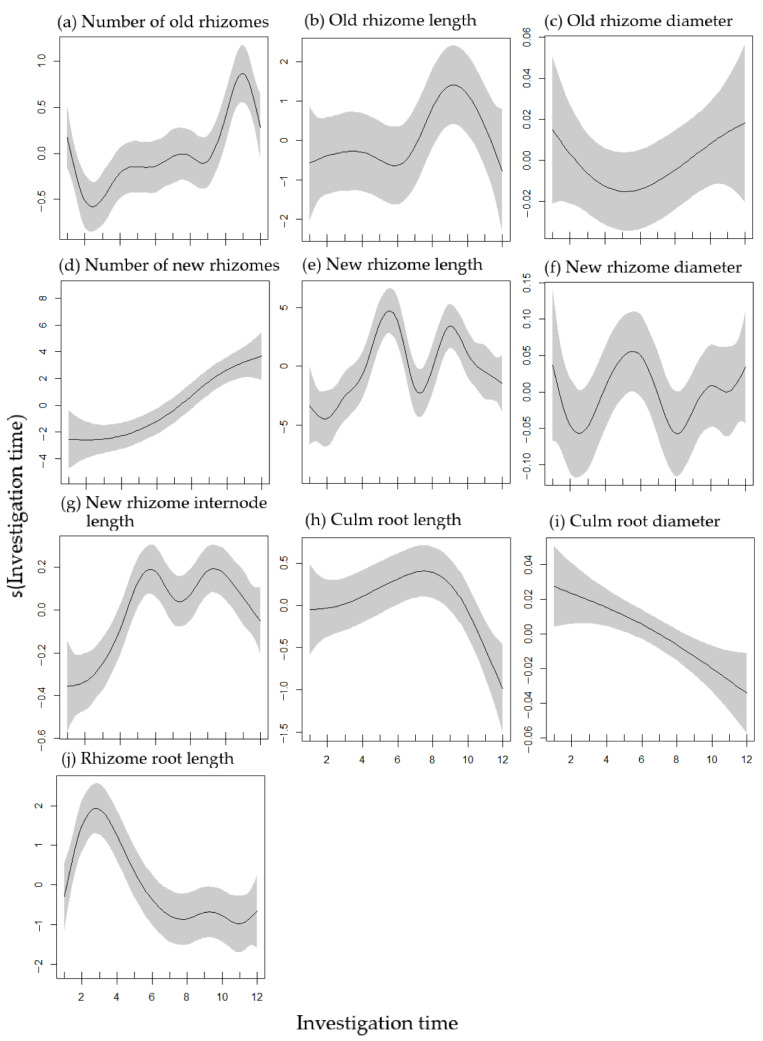
The plots of the GAMs smooth function for indicating the effects of investigation time on the number of old rhizomes (**a**), old rhizome length (**b**), old rhizome diameter (**c**), number of new rhizomes (**d**), new rhizome length (**e**), new rhizome diameter (**f**), new rhizome internode length (**g**), culm root length (**h**), culm root diameter (**i**) and rhizome root length (**j**). The grey ribbon shadow indicates the 95% confidence intervals of the fitted smoothers. The investigation times 1–12 in 2016 are 1 = 15 April, 2 = 9 May, 3 = 3 June, 4 = 27 June, 5 = 21 July, 6 = 12 August, 7 = 5 September, 8 = 23 September, 9 = 17 October, 10 = 10 November, 11 = 2 December, 12 = 26 December.

From the results of the generalized additive models (GAMs), it is obvious that the investigated eleven parameters showed diverse growth patterns along the growing period from April to December 2016 (Table 1; Figure 2). Non-significant dynamic growth changes were found in old rhizome length and old and new rhizome diameter during growing season (Figure 2b,c,f, Figure S1 and Figure S2 in the online supplementary data).

As the investigated time increased from one to twelve, the culm root diameter significantly reduced (degrees of freedom = 1.331, *p* < 0.01, Table 1, Figure 2i). However, the number of new rhizomes showed an opposite relationship with investigation time. The number of new rhizomes significantly increased from April to December (degrees of freedom = 2.712, *p* < 0.001, Figure 2d). In addition, as the light treatment continued, the gap between the control (CK) and treatments of the new rhizome number significantly widened, especially after the 9th investigation time (Figure S2 in the online supplementary data).

The new rhizome length and new rhizome internode length indicated a bimodal trend along the investigation time (Figure 2e,g). The new rhizome length and new rhizome internode length reached a peak at around the 6th and 9th survey times, and decreased to a trough at the 7th survey time. In addition, the value of the new rhizome length showed a lowest value at the 2nd survey time and a highest value at around the 6th survey time, whereas the value of the new rhizome internode length showed a lowest value at the 1st survey time and a highest value at around the 6th and 9th survey times.

The culm root length significantly correlated with investigation time, which increased from the first survey time and reached a peak at around the 8th survey time, then sharply declined (*p* < 0.05, Figure 2h, Figure S3 in the online supplementary data). The maximum value of the culm root length along the investigation time predicted from GAMs is the same as the actual value.

A significant change was found in the old rhizome number and rhizome root length along the survey time (*p* < 0.001). The GAMs predicted that the number of old rhizomes would decrease first, reach the minimum value at the 2nd sampling, then increase and reach a peak at the 11th sampling time. Thereafter, the number of old rhizomes would decrease again (Figure 2a). However, the rhizome root length tended to increase first and reached a peak at the 3rd survey time, and then rapidly decreased until the last survey time (Figure 2j). 

### 2.3. Effects of Light Intensity on Rhizome-Root System Growth

**Figure 3:**
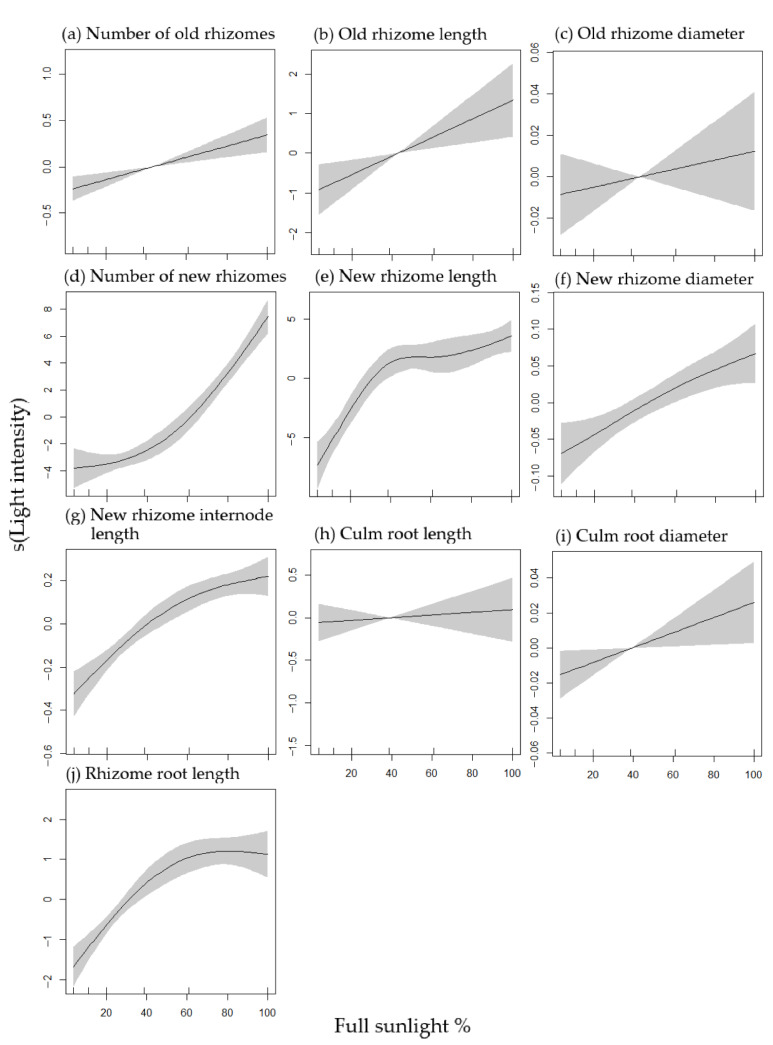
The plots of the GAMs’ smooth function for indicating the effects of light intensity on the number of old rhizomes (**a**), old rhizome length (**b**), old rhizome diameter (**c**), number of new rhizomes (**d**), new rhizome length (**e**), new rhizome diameter (**f**), new rhizome internode length (**g**), culm root length (**h**), culm root diameter (**i**) and rhizome root length (**j**). The grey ribbon shadow indicates the 95% confidence intervals of the fitted smoothers.

The results of the GAMs indicated that light intensity had a significant effect on the growth of the rhizome-root system of *P. pygmaeus* plants (Table 1, Figure 3). The relationships of old rhizome number vs. light intensity (*p* < 0.001), old rhizome length vs. light intensity (*p* < 0.05), and culm root diameter vs. light intensity (*p* < 0.05) were linear and positive (degree of freedom = 1.000, Figure 3a,b,i). In addition, the new rhizome diameter showed a significant, positive, and almost linear relationship with increasing light intensity (*p* < 0.05, degrees of freedom = 1.329, Figure 3f). All the above-mentioned four parameters were significantly increased as the light intensity increased from 0 to 100% full sunlight. 

The relationship of new rhizome number vs. light intensity was almost linear and positive (*p* < 0.001, degrees of freedom = 1.928). The new rhizome number significantly decreased as the light intensity was reduced from 100% full sunlight to around 40%, and then gradually decreased between 40% and 0% full sunlight (Figure 3d).

Non-linear, positive, and significant relationships were observed of new rhizome length vs. light intensity, new rhizome internode length vs. light intensity, and rhizome root length vs. light intensity (*p* < 0.001, Table 1 and Figure 3e,g,j). The new rhizome length rapidly increased from 0% to ca. 40% full sunlight, and the new rhizome internode length and rhizome root length dramatically increased from 0% to ca. 60% full sunlight, and all the three parameters then gradually increased until 100% full sunlight.

Non-significant relationships were observed of old rhizome diameter vs. light intensity and culm root length vs. light intensity (*p* > 0.05, Figure 3c,h).

### 2.4. A/U Ratio-Light Intensity and Investigation Time Analysis

**Figure 4:**
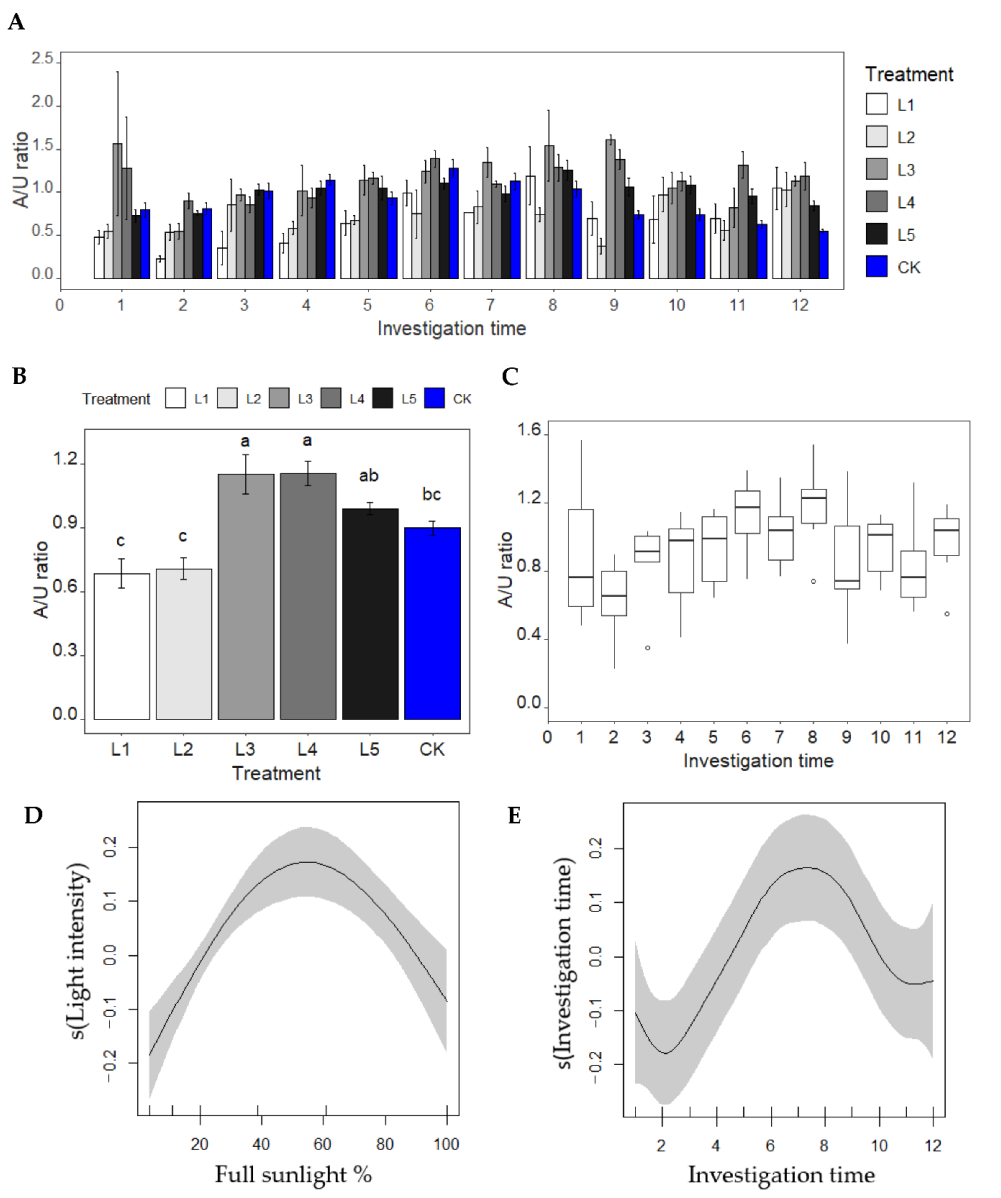
Effects of light intensity and investigation time on the ratio of aboveground to underground dry weight (A/U ratio). (**A**) The A/U ratio under different light intensity along the growing period from April to December; (**B**) average A/U ratio under different light treatment. A Different letter indicates significant differences between treatments at *p*  <  0.05 level; (**C**) box plots of A/U ratio under different investigation time; (**D**) the plots of the GAMs’ smooth function for illustrating the relationship of A/U ratio vs. light intensity; (**E**) the plots of the GAMs’ smooth function for illustrating the relationship of A/U ratio vs. investigation time.

The ratio of aboveground dry weight to underground dry weight (A/U ratio) was significantly affected by light intensity and investigation time (Figure 4). As the light intensity decreased to 38.76 and 20.25% full sunlight (L4 and L3), the A/U ratio was significantly increased, and then dramatically decreased under treatment L2 and L1 (11.25 and 3.87% full sunlight, Figure 4B). From the results of the GAMs, the A/U ratio presented a single peak curve along the light intensity and a prolonged investigation time (Figure 4D,E) with the highest value under around 55% of full sunlight and at around the 7th sampling time.

## 3. Discussion

### 3.1. Growth Characteristics of Rhizome-Root System

Bamboo rhizome-root systems are the basis of a bamboo forest’s growth and development [2]. Along the duration of the experiment from April to December, there were non-significant dynamic growth changes in the old rhizome length and old and new rhizome diameter of *P. pygmaeus*. However, the results of the GAMs predicted that the number of old and new rhizomes significantly increased from April to December. Under full sunlight (CK), the number of new rhizomes at the 9^th^ survey time was 3.1 times higher than that at the 8th survey time, which means that a lot of new rhizomes emerged between September and October (Figure S2 in the online supplementary data). This trend was similar with leaf dry biomass, which at the 9th survey time was 3.8 times larger than that at the 8th survey time [22]. We speculate that the growth of leaves, as the key component of plant’s photosynthetic apparatus [35,36,37], significantly affects new rhizome production but influences old rhizomes’ growth less. In other words, carbon used for new rhizome production might be mainly supported by leaf photosynthesis rather than stored non-structural carbonhydrates (NSCs), which is contrary to the proposal that stored NSCs are principally used for promoting new shoots growth [26,38]. The elongation of a new rhizome and its internodes showed a bimodal curve along the growing period, which reached the maximum value around late July and October. After the first peak (late July), the length of new rhizomes and new rhizome internodes significantly decreased until early of September. We speculate that during summer, *P. pygmaeus* adapts its traits to produce shorter rhizomes, which could shorten the nutrient and water transport distance in order to adapt to increasing temperatures and drought. After October, rhizome length decreases since *P. pygmaeus* enters the underground growth stage of bamboo shoots, which is similar to a previous study on *P. pubescens* [39]. However, the rhizome and culm root length increased first and reached a maximum value in June and September, respectively. Based on the results of our previous study, leaf- and aboveground-dry-biomass of *P. pygmaeus* plants were not fully developed when the length of new rhizomes, culm roots, and rhizome roots reached a peak [22]. Thus, we speculate that photosynthetic capacity is insufficient, and the new rhizome and root length growth might require a lot of NSCs transported from old rhizomes. This result is consistent with previous studies on *P. pubescens* that NSCs of leaves, branches, trunks, and rhizomes of attached mature bamboos were transformed and utilized into young bamboo growth [26,38]. During the growing season under full sunlight, the underground biomass (i.e., rhizome-root system) of *P. pygmaeus* plants was 1.11–1.95 times higher than the aboveground biomass. However, opposite results were found in other bamboo species, e.g., *Phyllostachys heterocycla* (Carr.) Mitford cv. Pubescens, *Phyllostachys praecox* C. D. Chu et C. S. Chao ‘Prevernalis’ and *Fargesia denudata* Yi, which indicated higher aboveground biomass than in the underground [40,41,42]. In addition, Umemura and Takenaka reported that the *P. pubescens* aboveground/underground biomass ratio is different at differing sites [43]. Specifically, moso bamboo growing on a mountainside with lower water content showed a significantly higher ratio of aboveground to underground biomass than that growing at mountain bases and near water flow. In addition, the culm roots significantly reduced their diameter from April to December, which was similar with culm that decreased its diameter from April until September [22]. However, it is still unknown why culm and culm root decrease its diameter during the growing period, which needs further anatomical and plant physiological study for this phenomenon to be revealed.

### 3.2. Effects of Light Intensity on Rhizome-Root System Growth

Light directly or indirectly influences virtually all morphological modifications occurring in both shoots and roots [6]. Previous studies confirmed that roots experienced significant changes in morphology and development in response to light, such as root length elongation, root cell elongation, lateral root formation, root nodule formation, nitrate uptake, and carbon assimilation [19,20,44,45,46]. Most of the mentioned studies used *Arabidopsis* as subject. However, the underlying morphological mechanisms that control how light influences bamboo rhizome-root growth remain poorly understood. Here, we report the effects of light illumination on *P. pygmaeus* rhizome-root system growth. We found that light intensity had a significant, positive, and linear/or almost linear impact on the number of old and new rhizomes, old rhizome length, new rhizome diameter, as well as the culm root diameter of *P. pygmaeus* plants. A nonlinear and positive relationship was found between light intensity and the listed three parameters, i.e., new rhizome length, new rhizome internode length, and rhizome root length. The value of the mentioned three parameters significantly increased from 0% to 40–60% full sunlight and then gradually increased until 100% of full sunlight. We found that when the light intensity went lower than ca. 40% of full sunlight, the GAMs predicted that the value of most parameters was lower than zero, e.g., the number of old and new rhizomes, old rhizome length, etc. Therefore, 40% of full sunlight might be the threshold for *P. pygmaeus* rhizome-root system growth. When light was lower than 40%, which is equal to an average light of 2232 lux, the underground growth of *P. pygmaeus* was inhibited.

Light, as one of the most important environmental factors, is not only involved in the shoot growth but also influences root growth and development. The aboveground- and underground-dry-biomass of *P. pygmaeus* were drastically reduced as the light intensity decreased [22]. However, the rate of decline was different. Under minor light control (60.76% of full sunlight), the ratio of aboveground to underground dry weight (A/U ratio) was not significantly different compared to being under full sunlight. Median light limitation (20.25–38.76% of full sunlight) reduced aboveground growth less than underground biomass, while extreme light limitation (<11.25%) reduced aboveground growth more than underground growth. This is consistent with our previous study on *P. pygmaeus* that the leaf/underground dry weight ratio showed a unimodal curve with the decreasing of light intensity [22]. Similar results were found in root/shoot ratio responses of *Eugenia uniflora* L., *Lactuca sativa* L. Var. youmaicai, *Lactuca sativa* L., *Fragaria × ananassa* Duch. cv. Benihoppe to changing light intensity [11,47,48,49]. Light can regulate COP1 (constitutively photomorphogenic 1) to stabilize some proteins (e.g., HY5, Elongated hypocotyl 5). Under deep shade, COP1-mediated light signaling can enhance the primary root elongation through modulating auxin transporter and activating PIN1 expression, increasing PIN1 and PIN2 localization on root-cell plasma membrane [50]. At the same time, HY5 moves from shoots to roots, regulating the expression of the auxin transporters PIN3 and LAX3 to coordinate shoot and root growth [51]. We speculate that under a median limitation (20.25–38.76% of full sunlight), *P. pygmaeus* might try to adjust its plasticity through increasing leaf area and stem height to optimize growth and performance in response to decreasing light intensity like other species [11,17,22,48]. The extreme light limitation (<11.25% full sunlight) might exceed the low light threshold for *P. pygmaeus* growth and result in A/U ratio dramatically reduction. Especially after October, leaf and new rhizome growth were significantly suppressed by low light, and the gap between control (CK) and treatments was significantly widened. A previous study on dwarf bamboos *Sinarundinaria nitida* (Mitford) Nakai found no difference in the ratio of leaf length to width under different light conditions, indicating that leaves in shade prefer to alter their size rather than change the shape [52]. In shade, woody dwarf bamboo can reduce its leaf vein density, which is the pathway for water flow and strongly relates to leaf hydraulic conductance [52,53,54]. Therefore, reduced leaf water supplied by lower vein density, lower stomatal density, and stomatal conductance could decrease the photosynthetic rate of *S. nitida* [52]. We speculate that the carbon assimilation products of *P. pygmaeus* might be reduced by decreasing light intensity, which consequently results in the reduction of rhizome-root biomass. 

## 4. Materials and Methods

### 4.1. Plant Materials, Soil, and Container

In March 2016, one-year-old *P. pygmaeus* plants from seeds were obtained from Baima Resource Nursery, Nanjing Forestry University, Nanjing, Jiangsu Province, China (119°9′15″ E, 31°36′49″ N). Soil was prepared by mixing nutrient soil and loess (1:2 volume ratio). The plastic containers with 51 cm outer-diameter, 44 cm inner-diameter, and 35 cm tall were selected.

### 4.2. Experimental Design and Light Intensity Recording

The pot experiment was performed in a greenhouse at Baima Resource Nursery. The top shed of the greenhouse was provided with two layers of inner and outer shading nets. A water curtain was set on one side of the greenhouse ventilation and an exhaust fan was set on the other side. In early May, we opened the outer shading net; in late May, the outer shading net and exhaust fan were opened; in mid-June, both of the inner and outer shading nets were opened; in early July, two layers of shading nets and water curtains were opened; in mid-October, the inner shading net was closed; and in mid-November, the outer shading net was also closed.

**Figure 5:**
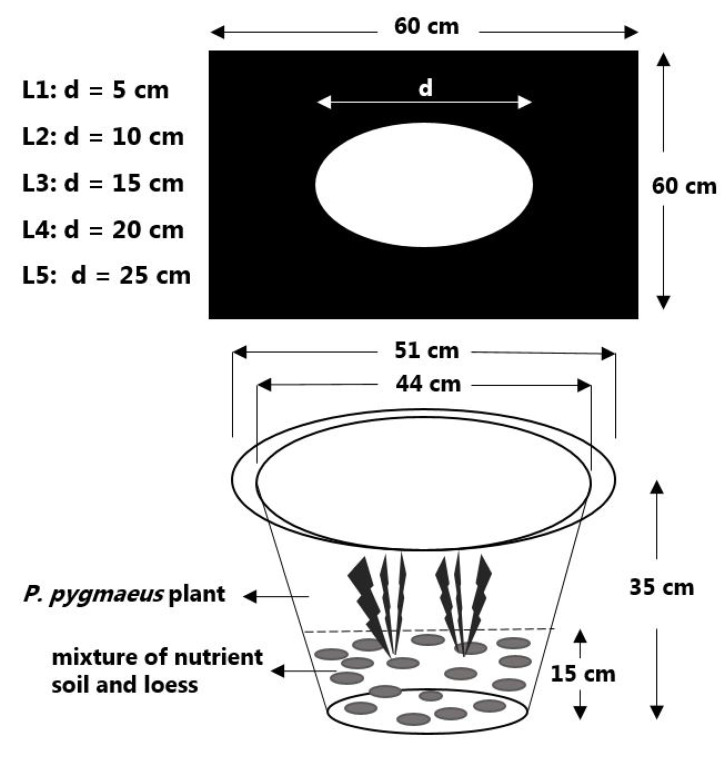
Experiment design and set-up. This drawing was obtained from Huang et al. [22].

The two-color plates were used to cover the pot to control light intensity, and the control plots (CK) were not covered by plates. The two-color plates were 60 cm × 60 cm in size and 0.5 cm in thickness. The tope side was wood color and opposite side was black. The light intensity was controlled by punching circular holes with different diameters in the center of the cover plates. The diameters of the punching circular holes were 5 cm, 10 cm, 15 cm, 20 cm, 25 cm, and assigned as treatment L1, L2, L3, L4, and L5 (Figure 5). The illuminance percentages of the pore diameter area to the inner diameter area of the pot were 1.29%, 5.16%, 11.62%, 20.66%, 32.28%, and 100% for treatment L1, L2, L3, L4, L5, and CK, respectively. Two bushes of one-year-old *P. pygmaeus* plants were transplanted into the center of each plastic pot in early March 2016. In total, 216 plants were transplanted with 36 replicates for each treatment.

One week after confirming the plants’ survival, two-color plates were used to cover the containers on 11 March 2016. The sampling and measurement were started on 15 April 2016, and then the next 11 samplings were conducted around every 25 days until December 2016. At each survey time, three pots of each treatment were randomly selected for direct measurement. The specific dates of the 12 investigation times in 2016 are 15 April, 9 May, 3 June, 27 June, 21 July, 12 August, 5 September, 23 September, 17 October, 10 November, 2 December, and 26 December. The specific sampling dates were equal to 36 d, 60 d, 85 d, 109 d, 133 d, 155 d, 179 d, 197 d, 221 d, 245 d, 267 d, and 291 d after the first day of light treatment, respectively.

During light treatment, a sunny and cloudless day was regularly selected each month to record the light intensity every half hour between 9:00 and 16:00 o’clock by a TES-1332A digital illuminance meter (TES Electrical Electronic Corp., Taipei). Three pots of each treatment were randomly selected to measure the light intensity. During measurement, the probe was set between the aperture of the cover plate and the canopy of the plant. When the value of the illuminance meter remained stable for more than three seconds, the value was recorded.

### 4.3. Growth Measurement

From April 2016 to December 2016, at each investigation time, various parameters to access the rhizome-root growth status were measured including: (i) old rhizome: number of old rhizomes, old rhizome length and old rhizome diameter, (ii) new rhizome: number of new rhizomes, new rhizome length, new rhizome diameter, and new rhizome internode length, (iii) culm root: length and diameter, (iv) rhizome root length.

After morphological measurement, the samples were dried at 80 °C for at least 72 h to a constant dry weight by a ventilated oven (Type: XMTD-8222, Jinghong Experimental Equipment Co., Ltd., Shanghai, China). After drying, the aboveground and underground parts were separated and weighed by an electronic balance (ME204/02, Mettler Toledo Company, Greifensee, Switzerland).

### 4.4. Statistical Analysis

Generalized additive models (GAMs) are a non-parametric extension of generalized linear models. The GAMs were used to access the correlation coefficient test of the joint effects of light intensity and investigation time on surveyed parameters:

(1) $$g\left(E(Y_{i}\right))=\beta_{0}+s_{1}(x_{i})+s_{2}(x_{i})+e_{i}$$

where g is a link function, $E(Y_{i})$ is the estimate for the responsible variable $Y_{i}$, $s_{1}$ is the smooth function of $x_{i}$ over different light treatments, $s_{2}$ is the smooth function of $x_{i}$ along investigation time, $x_{i}$ (i = 1, 2, 3,…, 12) are the explanatory variables, and they are number of new rhizomes, new rhizome length, new rhizome diameter, etc. $\beta_{0}$ is constant term and $e_{i}$ is error term. All calculations were conducted within the R environment using the “mgcv” package (version 3.6.3) [55].

## 5. Conclusions

In summary, this study provides a solid basis for understanding *P. pygmaeus* rhizome-root system growth patterns, and its growth responses to different light intensity. The results demonstrate that along the growing period from March to December, eight of the eleven studied rhizome-root parameters showed significant variability and diverse growth patterns. In addition, decreasing light intensity significantly reduced *P. pygmaeus* rhizome-root system growth. As the increasing of light intensity from 0% to 100% full sunlight, the number of old and new rhizomes, old rhizome length, new rhizome diameter and culm root diameter significantly and linearly/or almost linearly increased. When light availability was lower than 40% full sunlight, the new rhizome length significantly decreased; while when light intensity was lower than 60% full sunlight, the new rhizome internode length and rhizome root length were drastically reduced. Based on the prediction value of GAMs, 40% full sunlight (equal to average light of 2232 lux) might be the threshold for *P. pygmaeus* rhizome-root system growth. When light was lower than 40% full sunlight, the rhizome-root system growth of *P. pygmaeus* was inhibited. The A/U ratio showed a unimodal curve with decreasing light intensity. Under median limitation (20.25–38.76% of full sunlight), *P. pygmaeus* could try to adjust its plasticity through increasing stem height to optimize growth and performance. However, extreme light limitation (<11.25% full sunlight) might exceed its low light threshold and significantly reduce A/U ratio. Detailed analyses on how bamboo culms and rhizomes-roots coordinate their responses to light through light-signaling components and pathways need further investigation.

## Figures and Tables

**Table 1:** Results from the generalized additive models (GAMs) in explaining the influence of light intensity and investigation time on the rhizomes’ and roots’ growth of *P. pygmaeus* plants. *** *p <* 0.001; ** *p* < 0.01; * *p* < 0.005.

Parameters	Independent Variable	Degrees of Freedom	*F* Value	*P*r (>ǀtǀ)	*R* ^2^ *_adj_*
Number of old rhizomes	*S* (light intensity)	1.000	13.45	<0.001 ***	0.134
	*S* (invesitigation time)	7.504	5.85	<0.001 ***	
Old rhizome length	*S* (light intensity)	1.000	8.36	<0.05 *	0.038
	*S* (invesitigation time)	4.233	1.68	0.134	
Old rhizome diameter	*S* (light intensity)	1.000	0.75	0.388	0.004
	*S* (invesitigation time)	2.140	1.28	0.394	
Number of new rhizomes	*S* (light intensity)	1.928	74.57	<0.001 ***	0.418
	*S* (invesitigation time)	2.712	12.08	<0.001 ***	
New rhizome length	*S* (light intensity)	3.043	23.11	<0.001 ***	0.345
	*S* (invesitigation time)	8.107	5.70	<0.001 ***	
New rhizome diameter	*S* (light intensity)	1.329	7.74	<0.05 *	0.071
	*S* (invesitigation time)	6.171	1.38	0.230	
New rhizome internode length	*S* (light intensity)	1.825	23.90	<0.001 ***	0.292
	*S* (invesitigation time)	6.302	6.79	<0.001 ***	
Culm root length	*S* (light intensity)	1.000	0.25	0.617	0.034
	*S* (invesitigation time)	3.068	4.34	<0.05 *	
Culm root diameter	*S* (light intensity)	1.000	5.00	<0.05 *	0.029
	*S* (invesitigation time)	1.331	5.12	<0.01 **	
Rhizome root length	*S* (light intensity)	1.913	24.91	<0.001 ***	0.203
	*S* (invesitigation time)	5.761	7.17	<0.001 ***	
Ratio of aboveground dry weight to underground dry weight (A/U ratio)	*S* (light intensity)	1.965	14.15	<0.001 ***	0.118
	*S* (invesitigation time)	5.072	3.85	<0.001 ***	

## Data Availability

Not applicable.

## Supplementary Materials

The following supporting information can be downloaded at: https://www.mdpi.com/article/10.3390/plants11172204/s1, Figure S1: The plots of (A) number of old rhizomes, (B) old rhizome length and (C) old rhizome diameter under six different light intensity along the investigation time; Figure S2: The plots of (A) number of new rhizomes, (B) new rhizome length, (C) new rhizome diameter, and (D) new rhizome internode length under six different light intensity along the investigation time; Figure S3: The plots of (A) culm root length, (B) culm root diameter, and (C) rhizome root length under six different light intensity along the investigation time.
