# Supplementary material for: Growth and Development Responses of the Rhizome-Root System in Pleioblastus pygmaeus to Light Intensity
Source: Plants (Basel). 2022 Aug 25;11(17):2204. doi: 10.3390/plants11172204 (PMC9459886; doi:10.3390/plants11172204)
Supplement: Supplementary file 1 [file plants-11-02204-s001.zip › plants-1804570-supplementary.pdf]

Legends of supplementary figures

Figure S1. The plots of (A) number of old rhizomes, (B) old rhizome length, and (C) old rhizome diameter under six different light intensity along the investigation time. The error-bar indicates the standard error of six replicates. The illuminance percentages of the pore diameter area to the inner diameter area of the pot in each treatment were 1.29% (L1), 5.16% (L2), 11.62% (L3), 20.66% (L4), 32.28% (L5), and 100% (CK). The average light intensity of different treatments is L1 = 3.87%, L2 = 11.25%, L3 = 20.25%, L4 = 38.76%, L5 = 60.76%, and CK = 100% of full sunlight. The investigation times 1–12 in 2016 are 1 = 15 April, 2 = 9 May, 3 = 3 June, 4 = 27 June, 5 = 21 July, 6 = 12 August, 7 = 5 September, 8 = 23 September, 9 = 17 October, 10 = 10 November, 11 = 2 December, 12 = 26 December.

Figure S2. The plots of (A) number of new rhizomes, (B) new rhizome length, (C) new rhizome diameter, and (D) new rhizome internode length under six different light intensity along the investigation time. The error-bar indicates the standard error of six replicates. The information of the light intensity and investigation time please see the figure caption of Figure S1.

Figure S3. The plots of (A) culm root length, (B) culm root diameter, and (C) rhizome root length under six different light intensity along the investigation time. The error-bar indicates the standard error of six replicates. The information of the light intensity and investigation time please see the figure caption of Figure S1.

Figure S1.

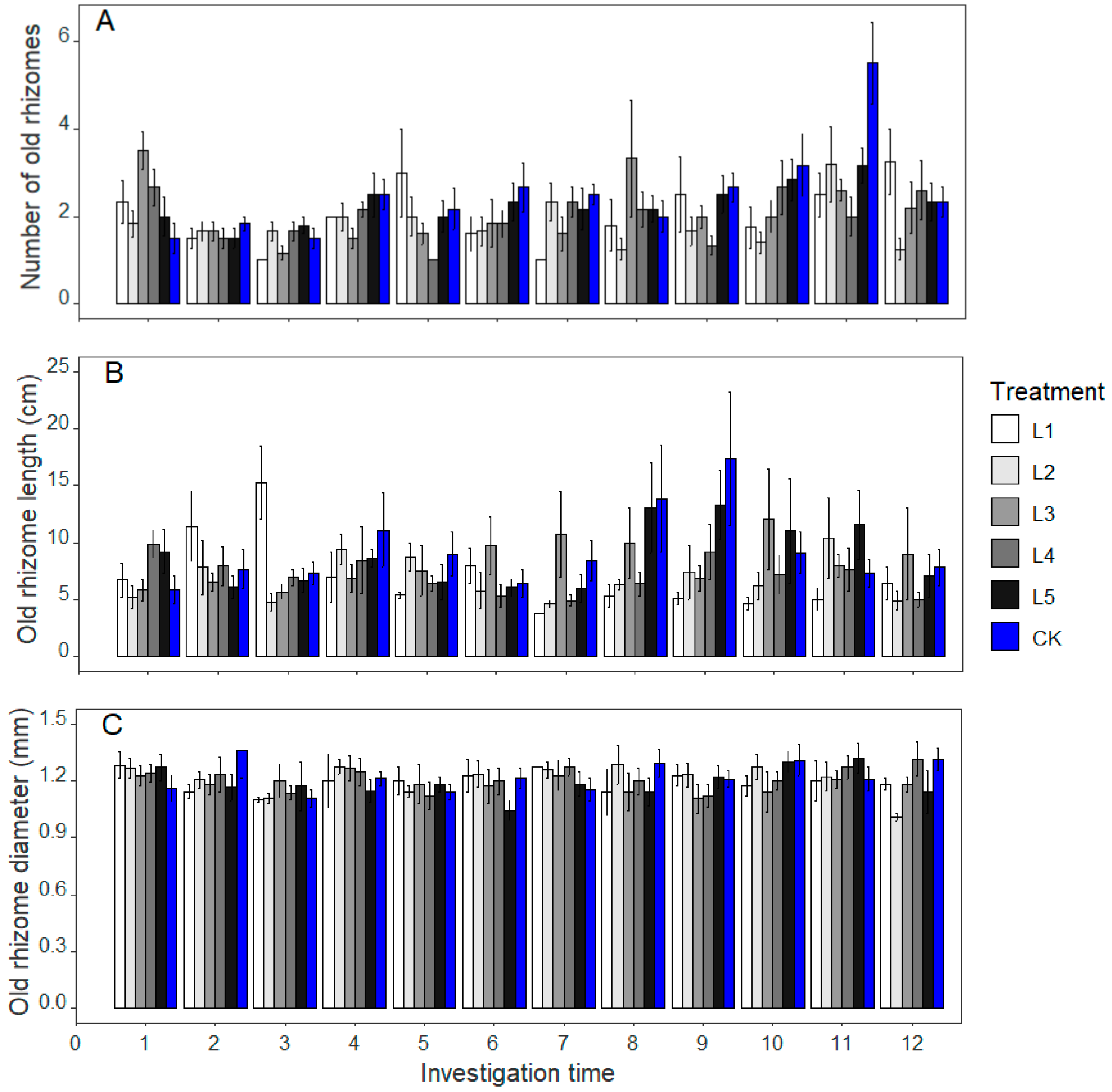
A
Number of old rhizomes
B
Old rhizome length (cm)
C
Old rhizome diameter (mm)
Investigation time
Treatment
L1
L2
L3
L4
L5
CK

Figure S2.

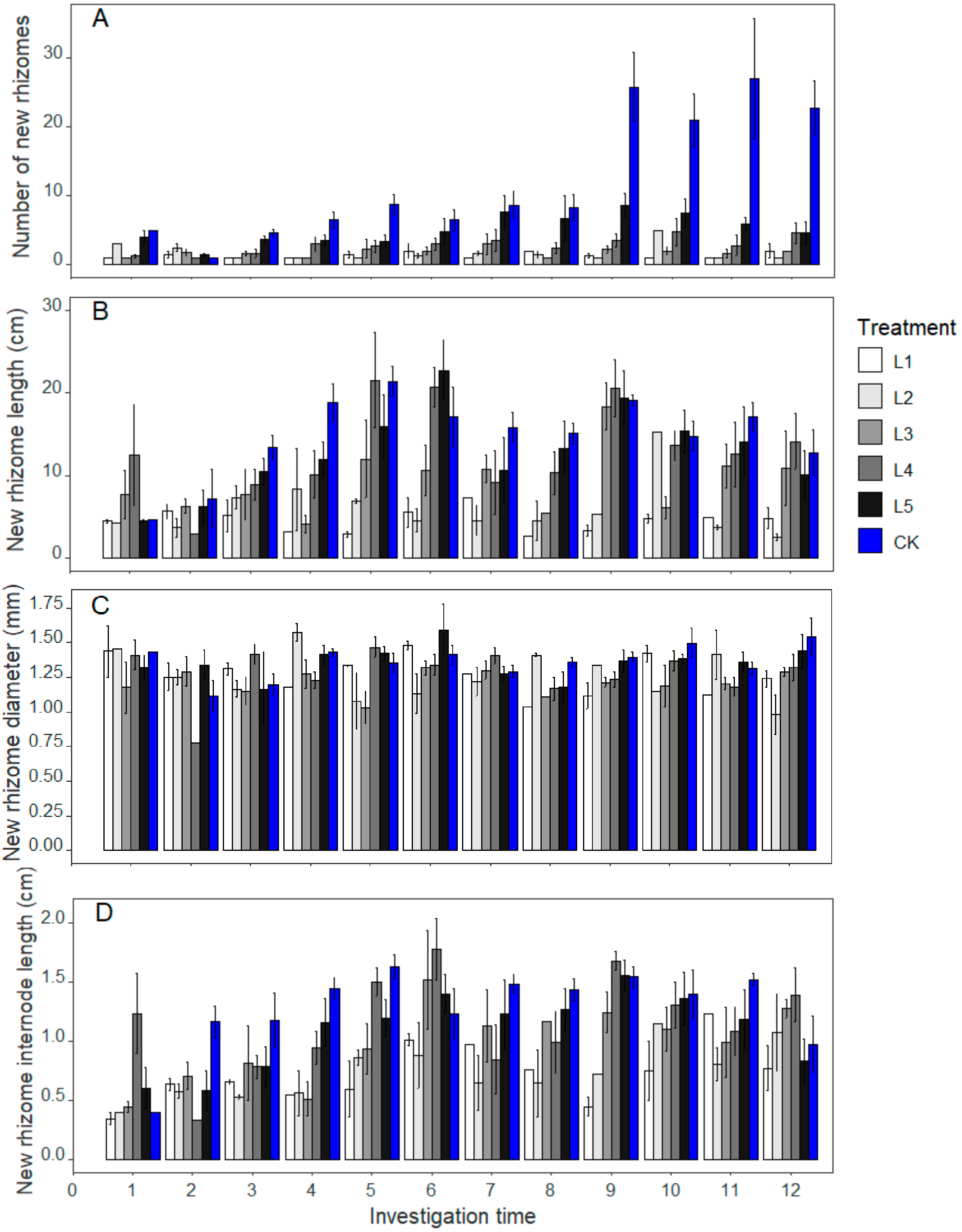
A
B
C
D
Number of new rhizomes
New rhizome length (cm)
New rhizome diameter (mm)
New rhizome internode length (cm)
Investigation time
Treatment
L1
L2
L3
L4
L5
CK
0
1
2
3
4
5
6
7
8
9
10
11
12

Figure S3.

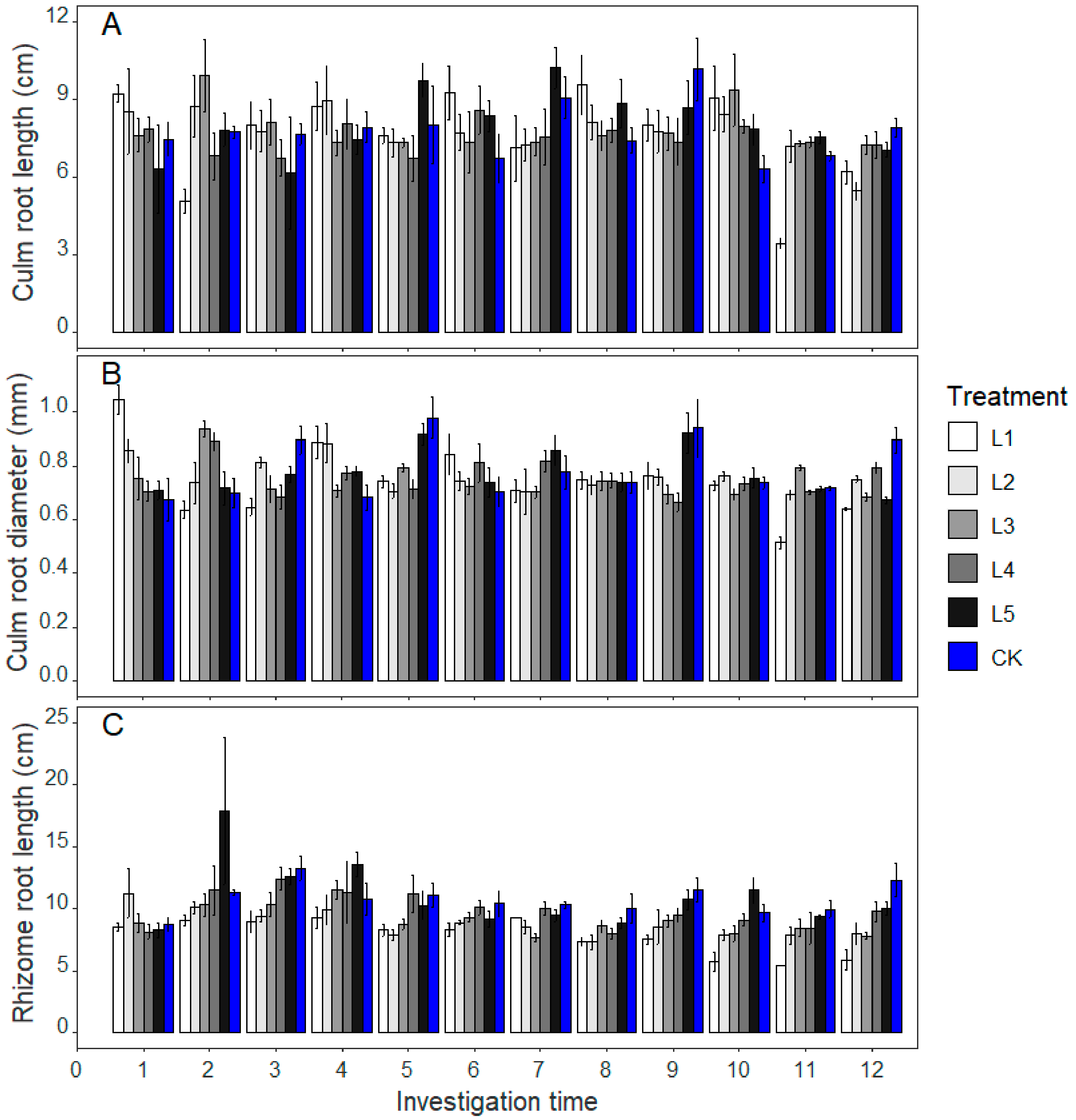
A
B
C
Culm root length (cm)
Culm root diameter (mm)
Rhizome root length (cm)
Investigation time
Treatment
L1
L2
L3
L4
L5
CK
